# Entropy estimation in acetylcholinesterase-donepezil interaction through topological indices: a graph theoretical perspective

**DOI:** 10.3389/fchem.2026.1794525

**Published:** 2026-05-20

**Authors:** K. B. Gayathri, S. Roy, K. Jyothish

**Affiliations:** Department of Mathematics, School of Advanced Sciences, Vellore Institute of Technology, Vellore, India

**Keywords:** acetylcholinesterase, donepezil, drug design, entropy, topological indices

## Abstract

In this work, we investigate the molecular interaction between acetylcholinesterase (AChE) and the Alzheimer’s medication donepezil using a graph-theoretical approach. We have presented the molecular graphs of AChE, donepezil, and their docked complex and calculated a set of degree-based topological indices, along with their respective Shannon entropy values. The entropy values measure the structural complexity and information content integral to each molecular graph. The study indicated a uniform growth of entropy for all indices in the docked complex over the unbound protein and ligand, signifying an enhancement in its topological disorder and edge-type variability with binding. It is interesting to see that indices such as the First Zagreb Index, Atom-Bond Connectivity Index, and Randić Index were successful in capturing this trend. This entropy-based assessment offers a solid and computationally efficient method for defining biomolecular interactions and providing insights into binding-induced structural reorganization. The results emphasize the value of topological entropy as a predictive tool for drug-target interaction profiling and structure-based drug design. Thus, the present study offers a computational approach to comprehend and forecast the energetics of a particular protein-ligand binding, which is a crucial objective in drug development and biophysical chemistry.

## 1 Introduction

Alzheimer’s disease is a progressive neurological illness that gradually impairs a person’s ability to learn, organize, think, and remember. It is induced by distinct molecular alterations in brain cells. Researchers from all around the world are looking for causes as well as preventative, diagnostic, therapeutic, and curing measures (Knopman et al., 2021; Reitz et al., 2011; Smith, 1998). The cholinergic enzyme acetylcholinesterase (AChE) is responsible for hydrolyzing the neurotransmitter acetylcholine (Soreq and Seidman, 2001). Acetylcholine has a significant impact on memory, learning, attention, and involuntary muscular action (Hasselmo, 2006). The patient develops Alzheimer’s disease as a result of the breakdown of this neurotransmitter.

Donepezil, a selective acetylcholinesterase inhibitor used to treat Alzheimer’s disease, is sold commercially under the name Aricept. As a centrally acting reversible acetylcholinesterase inhibitor, donepezil prevents acetylcholine from being hydrolyzed. Research on the development of donepezil was launched at Eisai in 1983, and in 1996, the US Food and Drug Administration approved the medication under the brand name Aricept (Dooley and Lamb, 2000; Donepezil, 2005; Tsuno, 2009; Cacabelos, 2007). The donepezil-acetylcholinesterase complex forms when donepezil occupies the catalytic anionic site (CAS) and the peripheral anionic site (PAS) of acetylcholinesterase. Strengthening pharmacological efficacy and improving drug design strategies requires an understanding of the molecular interaction between AChE and donepezil, especially from a thermodynamic and structural perspective.

Entropy is a key tool in understanding the drug-receptor interaction since it accounts for the disorder and flexibility of molecular systems. Quantitative estimation of the change in entropy during ligand binding provides important information regarding molecular recognition, stability, and conformational dynamics. The traditional approaches to entropy estimates, such as statistical mechanics-based models and molecular dynamics simulations, generally ask for complex parameterization and significant processing resources. By representing molecules as graphs and encoding their topological characteristics as topological indices (TIs), graph theory offers a competing, more intuitive, yet mathematically dynamic approach (Houck, 2020; Trinajstić, 2018).

Implementing graph theory, a discrete branch of mathematics, to chemistry, the field of chemical graph theory emerged. Chemical graphs assist in the representation of chemical structures in cheminformatics. In chemical graph theory, a molecule is examined by taking the atoms as vertices and the bonds as edges (Balaban, 1985; Bonchev, 2018). Topological indices are quantitative descriptors obtained from the graph structure, capturing features such as branching, molecular size, cyclicity, and atomic connectivity. These indices have demonstrated strong correlation with numerous physicochemical, thermodynamic, and pharmacokinetic characteristics of chemical compounds (Basak et al., 1987; Basak et al., 2002; Gutman, 2013; Rouvray, 1987).

Graph entropy serves as a vital function in chemical graph theory by providing information regarding the structural properties of molecular graphs. Entropy, often referred to as information entropy, was initially developed as a way to quantify the topological complexity and organization of chemical molecules. It is a key metric for assessing the stability and complexity of molecular networks. Structural information entropy has also been applied to assess the configuration and variability of multifarious graph-based systems, such as complex networks (Dehmer and Mowshowitz, 2011; Omar and Plapper, 2020; Simonyi, 1995). Among the several techniques used to investigate molecular structure, entropy based methods have proven very helpful in characterizing and quantifying structural patterns in chemical and biological systems. Many entropy formulations based on Shannon’s theory have been developed to evaluate graph topology; these often rely on basic invariants like vertex degree, edge distribution, interatomic lengths, and overall connectivity (Bein, 2006).

In the present work, we explore the molecular structure of acetylcholinesterase, donepezil, and their docked complex by calculating a series of topological indices. These are algebraic fingerprints of molecular architecture. Based on this, we analyze the corresponding entropy values to identify how structural order and complexity change upon binding. By characterizing entropy changes in individual molecules and their complex interactions, we provide new perspectives on topological and thermodynamic processes governing molecular recognition and stability.

It is important to have a firm understanding of the binding site in the complex to identify a new ligand or to improve an existing one to enhance ideal interactions with the target protein to obtain a better drug (Singh and Warshel, 2010; Mitra, 2009). This research provides a new graph-theoretical method for estimating entropy in biomolecular interactions that is independent of large-scale simulations or empirical models. Our method can be extended to other protein-ligand complexes and can potentially aid rational drug design by highlighting entropically favorable contacts. Through the combination of chemical graph theory and thermodynamic reasoning, this work adds to the expanding interdisciplinary community of mathematical chemoinformatics and helps to illuminate the structural principles of drug-target recognition.

The current study emphasizes the molecular-level characterization of protein-ligand interactions; however, it is crucial to acknowledge that these interactions represent the initial phase of a broader series of biological processes. Even though binding events happen quickly, the therapeutic effects that follow often involve slower, system-wide changes, like changes in the structure and function of biological systems. In this context, recent research has underscored the significance of physiological processes, such as lymphatic regulation and nitric oxide-mediated signaling, in sustaining homeostasis and affecting recovery mechanisms. The suggested graph-theoretical entropy framework is a basic tool that can be used for more complex biological systems, such as membrane receptor complexes and large-scale interaction networks. It is efficient to compute and is based on structure. These kinds of extensions might help connect molecular interactions to later physiological responses, helping us better understand how drugs work and how biomolecules behave (Davis and Bertram, 2025; Ohhashi et al., 2023).

## 2 Theoretical background

Graph theory offers a very powerful mathematical framework to represent molecular structures as graphs. Several topological indices have been developed in chemical graph theory to represent molecular structure in numerical form. These indices are particularly useful for determining entropy and are crucial tools for molecular complexity study. Let G be a simple connected graph with vertex set V(G) and edge set E(G) . Then δ(s) is the degree of vertex s , that is, the number of edges incident to s . For a molecular graph G , the general equation of a topological index based on vertex degrees is commonly defined as a function over the graph’s edges:

$$TI\left(G\right)=\underset{uv\in E\left(G\right)}{\sum }f\left(\delta \left(s\right),\delta \left(t\right)\right)\;,$$

where f(δ(s),δ(t)) is a real-valued function depending on the degrees of the end vertices of each edge. This function changes according to different topological indices (Ali et al., 2014; Estrada et al., 1998; Furtula et al., 2014; Furtula and Gutman, 2015; Gutman, 2021; Gutman et al., 2014; Gutman and Trinajstić, 2005; Randic, 1975; Shirdel et al., 2013; Vukičević and Furtula, 2009; Vukičević and Gaśperov, 2010; Zhong, 2012; Zhou and Trinajstić, 2009). The f(δ(s),δ(t)) values for a few selected topological indices used in this study are given below:

First Zagreb $\left[M_{1}\left(G\right)\right]$ : δ(s)+δ(t)

Second Zagreb $\left[M_{2}\left(G\right)\right]$ : δ(s)δ(t)

Hyper Zagreb [HM(G)] : ${\left(\delta \left(s\right)+\delta \left(t\right)\right)}^{2}$

Augmented Zagreb [AZ(G)] : ${\left[\frac{\delta \left(s\right)\delta \left(t\right)}{\delta \left(s\right)+\delta \left(t\right)-2}\right]}^{3}$

Randić Zagreb [R(G)] : $\frac{1}{\sqrt{\delta \left(s\right)\delta \left(t\right)}}$

Reciprocal Randić Zagreb [RR(G)] : $\sqrt{\delta \left(s\right)\delta \left(t\right)}$

Harmonic [H(G)] : $\frac{2}{\delta \left(s\right)+\delta \left(t\right)}$

Sum Connectivity [SC(G)] : $\frac{1}{\sqrt{\delta \left(s\right)+\delta \left(t\right)}}$

Geometric Arithmetic [GA(G)] : $\frac{2\sqrt{\delta \left(s\right)\delta \left(t\right)}}{\delta \left(s\right)+\delta \left(t\right)}$

Inverse Sum [IS(G)] : $\frac{\delta \left(s\right)\delta \left(t\right)}{\delta \left(s\right)+\delta \left(t\right)}$

Forgotten [F(G)] : $\delta {\left(s\right)}^{2}+\delta {\left(t\right)}^{2}$

Symmetric Division [SD(G)] : $\frac{\delta {\left(s\right)}^{2}+\delta {\left(t\right)}^{2}}{\delta \left(s\right)\delta \left(t\right)}$

Atom Bond Connectivity [ABC(G)] : $\sqrt{\frac{\delta \left(s\right)+\delta \left(t\right)-2}{\delta \left(s\right)\delta \left(t\right)}}$

Sombor [So(G)] : $\sqrt{\delta {\left(s\right)}^{2}+\delta {\left(t\right)}^{2}}$

For estimating the entropy from the edge-based allocation, we define the probability of each edge as:

$$p_{st}=\frac{f\left(\delta \left(s\right),\delta \left(t\right)\right)}{\underset{xy\in E\left(G\right)}{\sum }f\left(\delta \left(x\right),\delta \left(y\right)\right)}$$

Using Shannon’s entropy formula (Shanon, 1948), the entropy of the graph with respect to the considered topological index is expressed as:

$$\begin{array}{ll} H_{TI}\left(G\right) & =-\underset{st\in E\left(G\right)}{\sum }p_{st}\log p_{st}\\ & =-\underset{uv\in E\left(G\right)}{\sum }\frac{f\left(\delta \left(s\right),\delta \left(t\right)\right)}{\underset{xy\in E\left(G\right)}{\sum }f\left(\delta \left(x\right),\delta \left(y\right)\right)}\log\left(\frac{f\left(\delta \left(s\right),\delta \left(t\right)\right)}{\underset{xy\in E\left(G\right)}{\sum }f\left(\delta \left(x\right),\delta \left(y\right)\right)}\right)\\ & =\log\left(\underset{xy\in E\left(G\right)}{\sum }f\left(\delta \left(x\right),\delta \left(y\right)\right)\right)-\frac{1}{\underset{xy\in E\left(G\right)}{\sum }f\left(\delta \left(x\right),\delta \left(y\right)\right)}\underset{st\in E\left(G\right)}{\sum }f\left(\delta \left(s\right),\delta \left(t\right)\right)\log\left(f\left(\delta \left(s\right),\delta \left(t\right)\right)\right) \end{array}$$

This formulation allows for entropy estimation using any degree-based topological index by substituting the appropriate function f(δ(s),δ(t)) .

## 3 Materials and methods

The three dimensional crystallographic structures of acetylcholinesterase (AChE), donepezil, and the AChE-donepezil complex were downloaded from the Protein Data Bank (PDB) (RCSB, 2026). Namely:

- Acetylcholinesterase (AChE) was accessed via PDB ID 4PQ
- AChE-donepezil complex was taken from PDB ID 1EVE
- Donepezil was isolated as the ligand from the 1EVE complex

**FIGURE 1:**
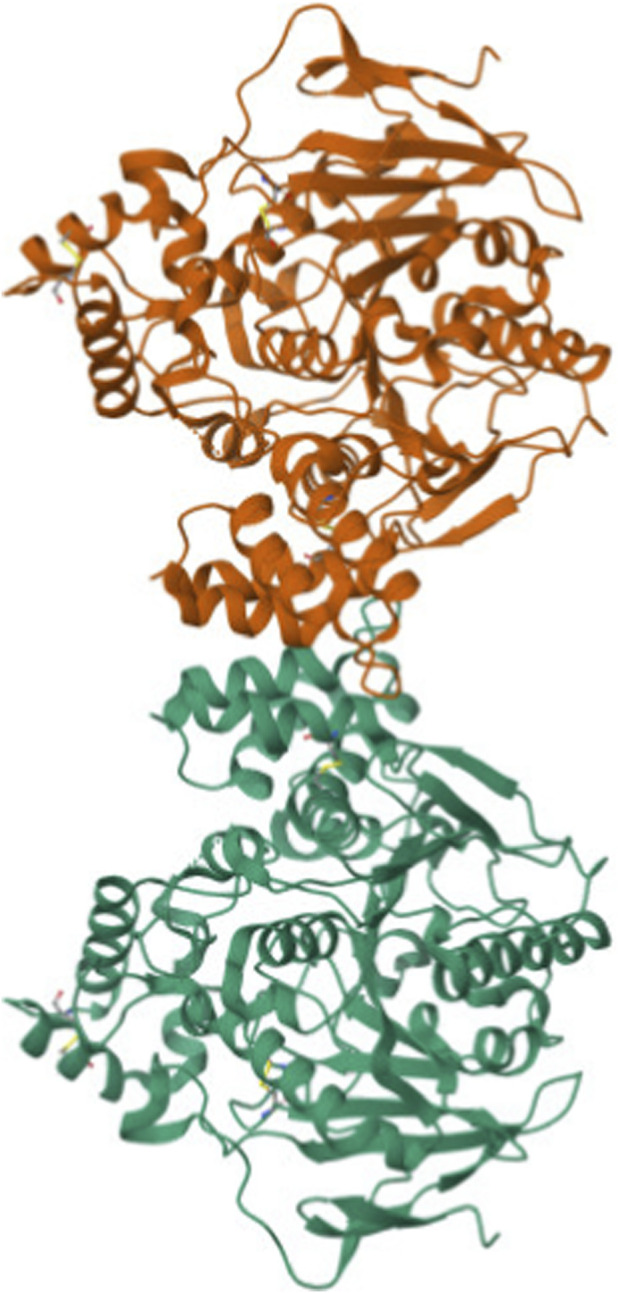
3D structure of acetylcholinesterase (PDB ID: 4PQE).

**FIGURE 2:**
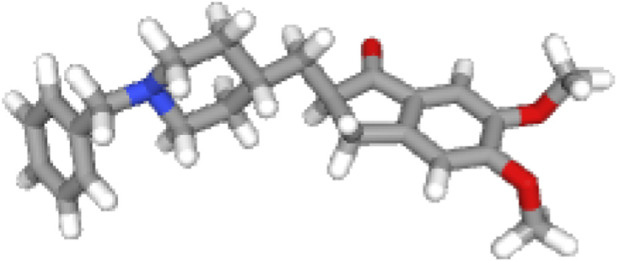
3D structure of Donepezil.

**FIGURE 3:**
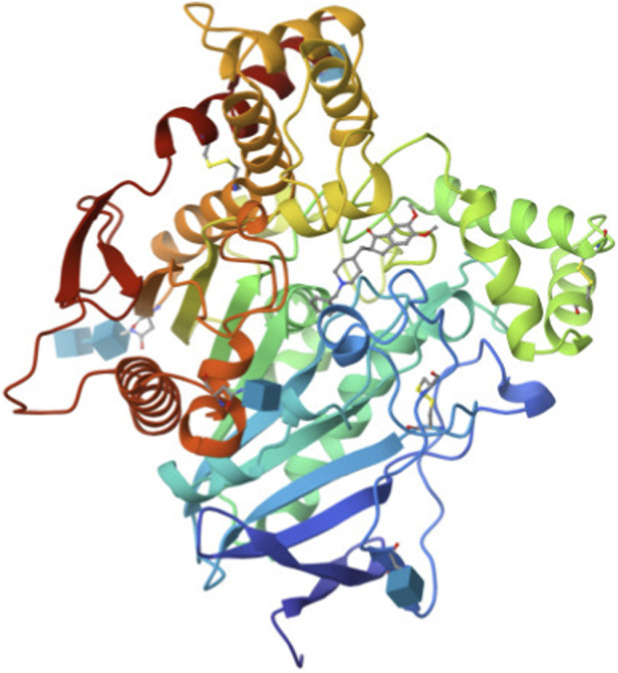
3D structure of acetylcholinesterase-donepezil complex (PDB ID: 1EVE).

Figures 1–3 shows the three dimensional structure of donepezil, acetylcholinesterase and donepezil-acetylcholinesterase complex respectively. The PDB files were downloaded, and these files were analyzed using MATLAB for graph-theoretical analysis.

MATLAB software was used for all further investigations. Each of the molecular structures was modeled into a molecular graph. The Cartesian coordinates of the atoms were extracted from the pdb files. If the Euclidean distance between any two atoms is less than a threshold of 1.9Å, then there exists a bond between the two (Bondi, 1964; Rappé et al., 1992). This approach was applied to all three molecular entities. An adjacency matrix is constructed for each compound by evaluating the Euclidean distance. From the adjacency matrix, the degree of each vertex was computed as the number of directly connected neighbors. To further analyze molecular interactions, based on the degrees of the atoms involved in each bond, the edge partitioning was performed. Based on the degree information, the previously mentioned topological indices were calculated using custom MATLAB code. These indices were calculated for the protein, ligand, and complex graphs individually to capture changes in molecular topology on interaction. The MATLAB code for calculating the edge partitioning, the total number of edges and the selected topological indices is given below.

*% === Step 1: Load PDB File ===*

pdbFile = ’structure.pdb’;
structure = pdbread(pdbFile);

*% === Step 2: Extract Atom Coordinates ===*

atoms = structure.Model.Atom;
numAtoms = length(atoms);
 coords = zeros(numAtoms, 3);
for i = 1:numAtoms
coords(i, :) = [atoms(i).X, atoms(i).Y, atoms(i).Z];
end
% === *Step 3: Construct Adjacency Matrix* ===
threshold = 1.9; % *Bond distance threshold*

adjMatrix = zeros(numAtoms);
 for i = 1:numAtoms
  for j = i+1:numAtoms
   dist = norm(coords(i,:) - coords(j,:));
   if dist < threshold
    adjMatrix(i,j) = 1;
    adjMatrix(j,i) = 1;
   end
  end
 end
% === *Step 4: Degree Vector* ===
deg = sum(adjMatrix, 2);
G = graph(adjMatrix);
E = G.Edges.EndNodes;
numEdges = size(E, 1);
% === *Step 5: Initialize Edge Partition Map* ===
partitionMap = containers.Map();
 for k = 1:numEdges
  u = E(k,1);
  v = E(k,2);
  du = deg(u);
  dv = deg(v);
  % *Sort the degree pair so (2,3) and (3,2) are the same*

  degPair = sort([du dv]);
  key = sprintf(’(%d,%d)’, degPair(1), degPair(2));
  % *Update the count in the partition map*

  if isKey(partitionMap, key)
   partitionMap(key) = partitionMap(key) + 1;
  else
   partitionMap(key) = 1;
  end
 end
% === *Step 6: Display the Edge Partition* ===
fprintf(’\n--- Edge Partition (Degree Pairs) ---\n’);
keysList = keys(partitionMap);
for i = 1:length(keysList)
 key = keysList{i};
 count = partitionMap(key);
 fprintf(’Edge Type %s : %d edges\n’, key, count);
end
% === *Step 7: Calculate Total Number of Edges* ===
totalEdges = sum(sum(adjMatrix)) / 2;
% === *Step 8: Display Result* ===
fprintf(’Total number of edges in the molecular graph: %d\n’, totalEdges);
% === *Step 9: Compute Indices* ===
M1 = 0; M2 = 0; RM2 = 0; HM = 0; AZ = 0;
R = 0; RR = 0; RRR = 0; H = 0; SC = 0;
GA = 0; IS = 0; F = 0; SD = 0; ABC = 0; So = 0;
for k = 1:numEdges
 u = E(k,1);
 v = E(k,2);
 du = deg(u);
 dv = deg(v);
 M1 = M1 + (du + dv);
 M2 = M2 + du * dv;
 RM2 = RM2 + (du - 1) * (dv - 1);
 HM = HM + (du + dv)^2^;
 AZ = AZ + ((du + dv) * sqrt(du * dv) / (du + dv - 2))^3^;
 R = R + 1 / sqrt(du * dv);
 RR = RR + sqrt((du + dv) / (du * dv));
 RRR = RRR + 1 / sqrt((du - 1) * (dv - 1));
 H = H + 2 / (du + dv);
 SC = SC + 1 / sqrt(du + dv);
 GA = GA + (2 * du * dv) / (du + dv);
 IS = IS + (du * dv) / (du + dv);
 F = F + du^2^ + dv^2^;
 SD = SD + (du^2^ + dv^2^) / (du * dv);
 ABC = ABC + sqrt((du + dv - 2) / (du * dv));
 So = So + sqrt(du^2^ + dv^2^);
end

Entropy was approximated from the distribution of the edge partitions obtained from the degree pairs. This method measures the structural information content and complexity of the molecular graph prior to and subsequent to ligand binding.

## 4 Numerical results

The partitioning of edges of the molecular graphs was done by grouping each edge based on the degree pair (δ(s),δ(t)) of the endpoints. This division indicates the distribution of local bonding environments and forms the basis of further entropy estimation. The edge type frequency of acetylcholinesterase, donepezil, and their complex is shown in Table 1.

**TABLE 1:** Edge partitioning based on vertex degrees in the molecular graphs of protein, ligand, and complex.

Edge type	Protein (AChE)	Ligand (donepezil)	Complex (AChE–Donepezil)
(1,2)	63	2	112
(1,3)	1110	1	1085
(2,2)	555	6	600
(2,3)	1809	18	1916
(3,3)	675	4	664
Total edges	4212	31	4377

**FIGURE 4:**
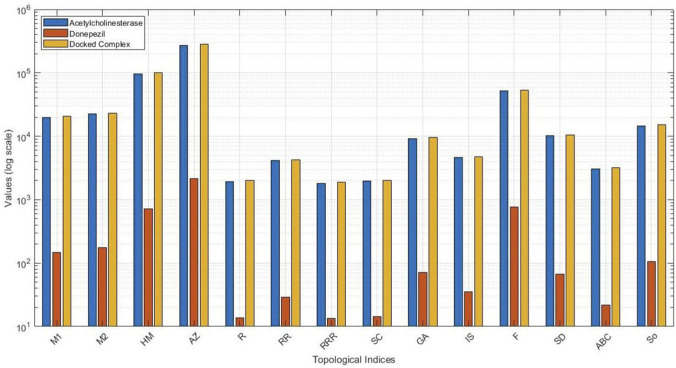
Clustered bar representation of comparison of topological indices for acetylcholinesterase, donepezil, and the docked complex.

In order to evaluate the structural complexity and bonding patterns in the molecular graphs, a series of degree-based topological indices were calculated for acetylcholinesterase, donepezil, and their docked complex. The indices represent various aspects of molecular connectivity, from simple degree summation to intricate geometric and harmonic relations. The obtained values are presented in Table 2, reflecting the impact of ligand binding on the protein’s topological characteristics. Figure 4 shows a clustered bar chart comparing the topological indices of acetylcholinesterase, donepezil, and the docked complex.

**TABLE 2:** Comparison of topological indices: acetylcholinesterase, donepezil, and docked complex.

Index	Acetylcholinesterase	Donepezil	Docked complex
$M_{1}$	19944	148	20640
$M_{2}$	22605	175	23351
HM	96732	724	99772
AZ	271069.22	2167.55	282930.05
R	1926.4277	13.6734	2009.1581
RR	4116.3952	29.3019	4281.2359
RRR	1823.1	13.3667	1904.9
SC	1953.4501	14.3375	2035.1013
GA	9225.6	71.37	9567.23
IS	4612.8	35.68	4783.62
F	51522	374	53070
SD	10237	67.33	10576
ABC	3072.4594	21.8679	3186.8421
So	14607	106.48	15103.92

**FIGURE 5:**
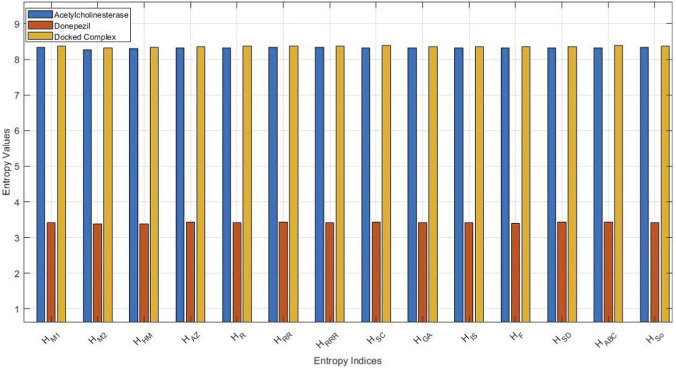
Comparison of entropy measures for acetylcholinesterase, donepezil, and the docked complex.

The Shannon entropy values calculated for a set of degree-based topological indices give a numerical estimate of molecular structural complexity in the acetylcholinesterase(AChE), donepezil, and their docked complex molecular graphs and are showcased in Table 3 and Figure 5, which gives a clustered bar graph for the same. For all the chosen indices, such as the First Zagreb index $\left(M_{1}\right)$ , Augmented Zagreb index (AZ) , Randic index (R) , and Atom Bond Connectivity index (ABC) , the docked complex had a higher entropy value than the individual components. For instance, the entropy corresponding to the ABC index rose from 8.3430 (AChE) and 3.4334 (donepezil) to 8.3816 in the complex, indicating an increase in edge type diversity and topological irregularity when the ligand binds. The rise indicates that molecular docking adds new edge configurations as well as degree pair distributions to the molecular graph, especially in and around the active site. The uniformly higher entropy values of the complex across indices suggest a higher level of structural information content and non-uniformity, typical of biologically active conformational states. The binding entropy can be calculated as:

$$\Delta H_{\text{binding}}=H_{\text{Complex}}-\left(H_{\text{Protein}}+H_{\text{Ligand}}\right)$$

**TABLE 3:** Entropy values of acetylcholinesterase, donepezil, docked complex, and the corresponding entropy change upon binding.

Entropy	Acetylcholinesterase	Donepezil	Docked complex	$\Delta H_{\text{binding}}$
$H_{M_{1}}\left(G\right)$	8.333578	3.421134	8.371539	−3.383173
$H_{M_{2}}\left(G\right)$	8.273253	3.382217	8.311702	−3.343768
$H_{HM_{2}}\left(G\right)$	8.298118	3.387829	8.335319	−3.350628
$H_{AZ}\left(G\right)$	8.313581	3.424277	8.353740	−3.384118
$H_{R}\left(G\right)$	8.325781	3.415171	8.363704	−3.377248
$H_{RR}\left(G\right)$	8.337860	3.428752	8.376324	−3.390288
$H_{H}\left(G\right)$	8.333421	3.417857	8.370945	−3.380333
$H_{SC}\left(G\right)$	8.342627	3.430177	8.380858	−3.391946
$H_{GA}\left(G\right)$	8.317402	3.417340	8.356166	−3.378576
$H_{IS}\left(G\right)$	8.317402	3.417340	8.356166	−3.378576
$H_{F}\left(G\right)$	8.312279	3.391533	8.348763	−3.355049
$H_{SD}\left(G\right)$	8.321907	3.428288	8.361182	−3.389013
$H_{\mathrm{ABC}}\left(G\right)$	8.343003	3.433406	8.381554	−3.394855
$H_{So}\left(G\right)$	8.337101	3.422160	8.374884	−3.384377

A negative entropy change, or $\Delta H_{\text{binding}}<0$ , indicates that binding makes the system more ordered. For every index, the computed entropy changes $\left(\Delta H_{\text{binding}}\right)$ are consistently negative, with values ranging from −3.34 to −3.39 . This consistent pattern indicates a substantial decrease in the entropy after binding of donepezil with acetylcholinesterase. Among the measurements, $H_{HM_{2}}\left(G\right)$ shows the least reduction (−3.3438) , whereas $H_{\mathrm{ABC}}\left(G\right)$ shows the biggest decline (−3.3948) . The magnitude of these values indicates that the binding event limits the system’s conformational freedom, resulting in a complex that is more ordered and stable. This type of entropy decrease characterizes robust and selective drug–receptor interactions. This work thus proves that entropy calculated from edge-partitioned topological indices is a reliable graph-theoretical descriptor for identifying interaction-induced perturbations in biomolecular systems. By evaluating and comparing the entropy variations of the AChE-donepezil complex with those of complexes formed by recently developed ligands, it is possible to make an accurate prediction about the binding efficiency and potential inhibitory performance of the new drugs. It hence has the potential for applications in molecular interaction profiling, ligand efficacy assessment, and rational drug design.

## 5 Conclusion

This is a novel work that shows the effectiveness of graph-theoretical entropy in studying the structural complexity of biomolecules and their interactions. In this paper, by modeling acetylcholinesterase, donepezil, and their docked complex as molecular graphs and analyzing them using degree-based topological indices, we measured the alterations in structural organization upon ligand binding. The rise in Shannon entropy in all indices for the docked complex reflects increased topological irregularity and heterogeneity, indicative of a significant structural reorganization caused by molecular binding. This observation was further supported through the partitioning of edges that showed the restructuring of degree-based edge types in the complex. These observations underscore the utility of entropy based on topological descriptors as a sensitive and computationally efficient measure to identify binding-induced perturbations. The strategy not only enhances the theoretical insight into molecular interactions but also provides useful applications in rational drug design, ligand optimization, and computational pharmacology.

## Data Availability

The original contributions presented in the study are included in the article/supplementary material, further inquiries can be directed to the corresponding author.
